# Warfarin-Induced Skin Necrosis Despite Enoxaparin Bridging Therapy

**DOI:** 10.7759/cureus.20883

**Published:** 2022-01-02

**Authors:** Mohamed Wali, Muhammad T Latif, Mary Lockwood, Ayman Saeyeldin, Carolina Borz-Baba

**Affiliations:** 1 Internal Medicine, Frank H. Netter M.D. School of Medicine at Quinnipiac University, North Haven, USA; 2 Internal Medicine, Saint Mary’s Hospital, Waterbury, USA; 3 Internal Medicine, NewYork-Presbyterian Brooklyn Methodist Hospital, Brooklyn, USA

**Keywords:** enoxaparin, warfarin therapy, drug-induced hypersensitivity, drug rash, bridging anticoagulation, warfarin-induced skin necrosis

## Abstract

Skin necrosis is a rare albeit severe complication of warfarin use for anticoagulation, resulting in significant morbidity and mortality. Here, we present the case of a 58-year-old woman who developed erythema and pain in her left leg two weeks after initiation of warfarin therapy with concomitant early administration of heparin for a deep vein thrombosis and pulmonary embolism. Subsequently, the erythema progressed to skin necrosis, and a diagnosis of warfarin-induced skin necrosis (WISN) was suspected. Warfarin was discontinued, and unfractionated heparin was commenced. The simultaneous presence of eosinophilia pointed toward an associated drug-related hypersensitivity reaction. Dexamethasone was added to the medication regimen. The patient was discharged on a factor Xa inhibitor and prednisone.

Recognizing WISN is crucial in patients receiving anticoagulation. The diagnosis can be particularly challenging in cases when bridging anticoagulation has been previously completed. Early diagnosis and drug discontinuation are critical to ensuring a favorable prognosis. Steroids may also play a role in the treatment of this condition if an associated drug hypersensitivity is identified.

## Introduction

Warfarin is a vitamin K antagonist (VKA) that is frequently used in various clinical settings where anticoagulation is necessary. VKAs inhibit gamma-carboxylation and activation of clotting factors II, VII, IX, and X, leading to its anticoagulation effects. VKAs also inhibit gamma-carboxylation of anticoagulant proteins C and S [1], resulting in a transient procoagulant effect during the first few days of use. Microthrombi formation and endothelial damage can progress to skin necrosis [2]. Most commonly, this occurs in the first 10 days of starting warfarin [2]. However, recent case reports have described a late-onset presentation which can vary from months to years [2].

Warfarin-induced skin necrosis (WISN) has been estimated to develop in 0.01-0.1% of individuals on VKAs [2]. Patients at the highest risk include those with an underlying thrombophilia or those who have not been pretreated with a non-VKA anticoagulant.

Patients present initially with erythema and pain followed by the rapid development of petechiae and hemorrhagic bullae, which eventually leads to skin necrosis [3]. This condition is associated with significant morbidity and mortality, and, if suspected, the offending agent should be discontinued promptly [4].

## Case presentation

A 58-year-old woman with a recent diagnosis of left leg deep vein thrombosis (DVT) and saddle pulmonary embolism (PE) arrived at the emergency department for worsening edema and erythema of her left leg for the past two days. Two weeks prior to her current presentation, the patient had received inpatient anticoagulant therapy for her PE and DVT. The treatment regimen included three days of unfractionated heparin followed by two days of low-molecular-weight heparin (LMWH) along with 5 mg of warfarin. Warfarin was increased to 7.5 mg on the day of discharge as there was no increase in her international normalized ratio (INR). The patient was discharged after six days of hospitalization on 120 mg of enoxaparin twice daily (her weight at the time of discharge was 147.6 kg) and 7.5 mg of warfarin daily, with instructions to closely follow up with her primary care physician for INR checks and dosage adjustments. Her INR at discharge was 1.2. The patient stated she had complied with medication instructions, though it was unclear if she followed up with her primary care provider.

The patient’s medical and surgical history included diabetes mellitus type 2, depression, migraines, spinal stenosis, and an appendectomy. Besides warfarin, her home medications included duloxetine, topiramate, and gabapentin. She was not being treated for diabetes at the time of admission.

**Figure 1:**
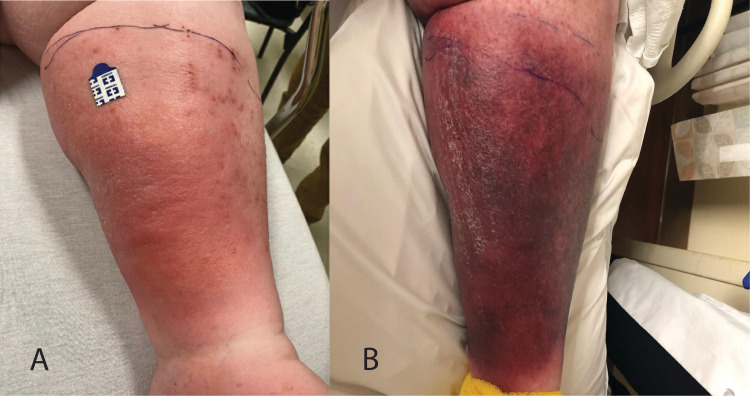
Early course of the left lower extremity erythema. A: Initial hospital presentation. B: Day three of hospital presentation.

On her current physical examination, the patient appeared comfortable. Her blood pressure was 128/64 mmHg, temperature was 98.7°F, heart rate was 102 beats per minute, and respiration rate was 24 breaths per minute. She had bilateral pitting edema, equal warmth on both legs, and erythema at the lower left leg (Figure 1, Panel A). There was tenderness on the posterior aspect of her left lower leg, without areas of fluctuance or collection. No mucosal lesions were noticed.

The initial laboratory results revealed white blood cells of 5.4 k/µL (normal range 4.0-10.5 k/µL), hemoglobin 12.3 g/dL (12.5-16.0 g/dL), platelets 237 k/µL (150-450 k/µL), sodium 138 mEq/L (136-145 mEq/L), potassium 3.8 mEq/L (3.5-5.1 mEq/L), glucose 127 mg/dL (70-105 mg/dL), creatinine 1.1 mg/dL (0.7-1.3 mg/dL) similar to baseline, blood urea nitrogen 17 mg/dL (7-25 mg/dL), total bilirubin 0.5 mg/dL, alkaline phosphatase 85 U/L, aspartate transaminase 58 U/L, and alanine aminotransferase 46 U/L.

The patient received one dose of cephalexin and was admitted for observation under the medicine service with a possible diagnosis of cellulitis versus recurrent DVT despite a supratherapeutic INR of 3.9. An ultrasound venous duplex showed no DVT. On the next day, the patient developed a fever of 100.4°F, and vancomycin was started for suspicion of sepsis due to skin and soft tissue infection. Blood cultures were negative, and repeat INR was between 2 and 3, for which warfarin was restarted. On day three, she continued to have a relapsing and remitting fever. On physical examination, a dark petechial rash was noted (Figure 1, Panel B).

A complete blood count revealed increased segmented neutrophils as well as mild eosinophilia with an absolute eosinophil count of 700. The presence of negative blood cultures, absence of leukocytosis, and worsening cutaneous skin findings despite antibiotic treatment were inconsistent with an infectious process. After consulting with infectious disease, a decision was made to discontinue vancomycin and other home medications. The presence of eosinophilia and symptoms coinciding with vancomycin administration were concerning for a drug-related hypersensitivity reaction, and intravenous dexamethasone was prescribed.

**Figure 2:**
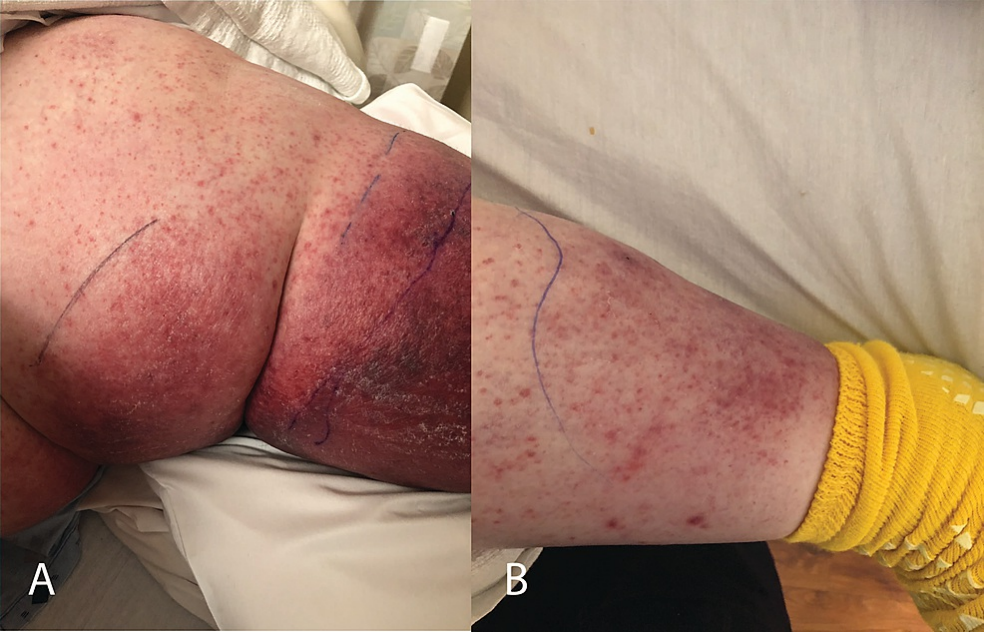
Erythema progression and macular rash development. A: Left lower extremity day four. B: Right lower extremity day four.

Over the next day, the erythema increased in size and progressed to blood-filled bullae (Figure 2A). The patient also began developing minor macular rashes over the abdomen (not pictured) and the previously unaffected right lower extremity (Figure 2B). No lymphadenopathy was appreciated on physical examination.

**Figure 3:**
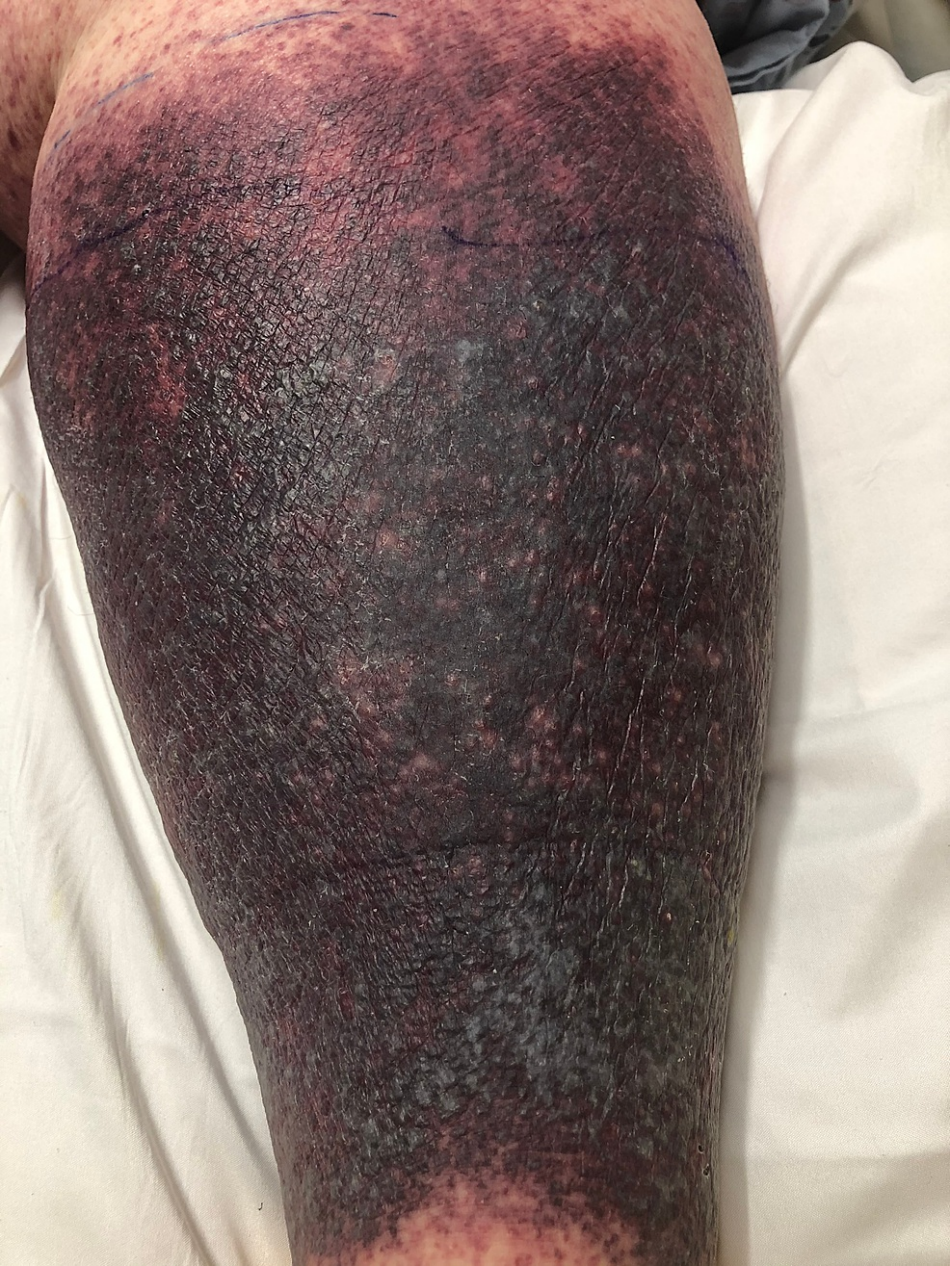
Left lower extremity discoloration.

**Figure 4:**
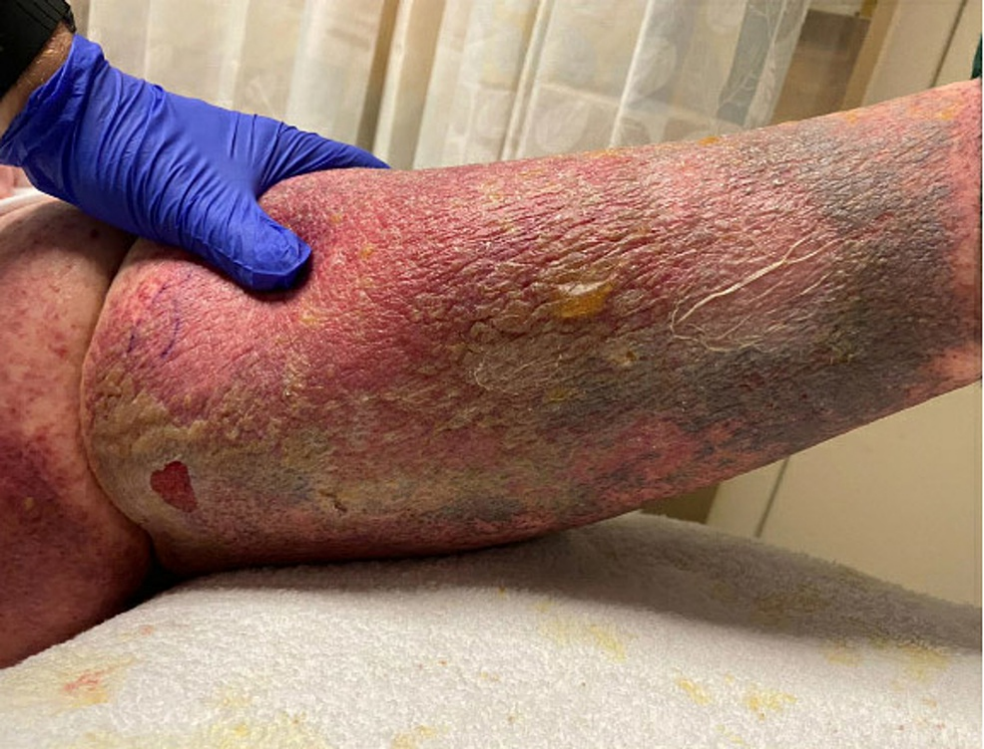
Left lower extremity rash improvement.

Warfarin was not initially discontinued as early suspicion for WISN was low due to proper bridging therapy and because the patient had a DVT/PE just three weeks prior. However, with the worsening progression of the rash, hematology-oncology was consulted on day six. Given the severe complications of WISN, discontinuation of warfarin was recommended even with an atypical presentation. Intravenous heparin was initiated. Subcutaneous 10 mg vitamin K and one unit of fresh frozen plasma were administered. Biopsy was discussed with infectious disease and hematology but not performed due to the severity of pain and potential irreversible additional wounds. Dermatology was not consulted during this hospitalization as management would likely have not changed. The bullous erythematous rash initially progressed to a discolored black rash (Figure 3), but after eight days of heparinization, the patient reported significant improvement in her left lower extremity pain and rash (Figure 4).

The patient’s maculopapular rash on her left lower extremity significantly improved, with other areas of erythema improved or resolved. New areas of yellow bullae with drainage and unroofed bullous lesions developed on the left lower extremity. Surgery and wound care were consulted, with surgical intervention deemed unnecessary but local wound care applied to enhance recovery. Wound care also recommended against biopsy due to the risk of iatrogenic non-healing ulcers. The patient was discharged on prednisone 40 mg with a tapering dose for four weeks and apixaban with counseling to follow up with her primary care physician, wound care, dermatology, and hematology for further management and observation.

## Discussion

WISN is caused by microvascular thrombosis followed by hemorrhage and necrosis of the dermis due to protein C deficiency in the presence of normal levels of vitamin K-dependent clotting factors and a possible direct toxic effect [5]. Hereditary protein C, protein S, and antithrombin III deficiency are the most common predisposing factors [6].

Female gender, obesity, perimenopausal age, viral infections, hepatic disease, large doses of warfarin, and drug interactions are also risk factors [7]. The progression of this disease begins with complaints of intense pain in the affected area and erythema, leading to ecchymosis, petechiae, discoloration, and hemorrhagic bullae. Subsequently, necrosis of the skin and subcutaneous tissue ensues [8]. Areas with large subcutaneous adipose tissues are common sites for WISN, including the breasts, buttocks, abdomen, thighs, and extremities. Our patient was a 59-year-old postmenopausal obese female with the same presentation of WISN described above on her left lower extremity as well as rashes on her abdomen and right lower extremity. In our patient’s case, she previously experienced DVT on the left lower extremity. The differential diagnosis could be very challenging as diverse warfarin-induced dermatological lesions have been reported from the classic “purple toe syndrome” to urticaria [9] or drug rash with eosinophilia and systemic symptoms (DRESS) [10]. Infectious causes such as necrotizing fasciitis, vasculitis, and disseminated intravascular coagulation (DIC) are frequently considered among the masqueraders of WISN [4]. The initial differential diagnosis in this patient included skin necrosis related to warfarin, a recurrence of her DVT, skin and soft tissue infection, atypical DRESS, and Stevens-Johnson syndrome (SJS). Venous Doppler of the lower extremity excluded a DVT. Cellulitis with fasciitis was unlikely considering the less severe clinical presentation with the absence of crepitus, absence of sepsis, and worsening of the skin findings, irrespective of the antibiotic treatment. SJS usually presents with a centripetal rash progressing to skin sloughing and associated with mucosal lesions, which was absent in our patient.

DRESS remained the most important differential diagnosis in this case. Typically, DRESS presents with rash, eosinophilia, and fever after weeks of commencing a medication. The unilaterally distributed erythema progressing to necrosis, the absence of lymphadenopathy, or other organ involvement (e.g., liver, lung, kidney) significantly decreased the possibility of DRESS in our patient; however, it did not exclude it completely. An accompanying hypersensitivity reaction was considered in our patient and steroids were added to the treatment regimen.

Although the diagnosis of WISN is mainly based on clinical grounds, the differential diagnosis can be challenging for practitioners, particularly in the incipient phase of this condition when erythema associated with fever suggests an infectious or a vasculitis process. The presence of eosinophilia could further complicate the interpretation of the cutaneous findings as hypersensitivity reactions are among other rare complications of warfarin treatment. Although not performed in our patient, biopsy can play a role in distinguishing WISN from other mimicking conditions such as DRESS as well as vasculitis, calciphylaxis, microembolization, DIC, or thrombotic thrombocytopenic purpura, among others.

Regarding prevention, the common practice includes the concomitant administration of heparin for five days to prevent the occurrence of WISN. Another crucial cautionary measure is to avoid prescribing a high loading dose of warfarin (≥10 mg) [11]. Our patient had received an appropriate duration of combined anticoagulant therapy for at least a week. WISN, despite prior heparinization, is extremely rare. To our knowledge, there is only one similar case report in the literature [8]. The scarcity of reports precludes us from estimating the probability or the pathogenesis of WISN in patients adequately co-anticoagulated with heparin.

Immediate withdrawal of warfarin is considered paramount when suspecting WISN, even when adequate heparin bridging therapy has been performed. Based on one previous study, discontinuation of warfarin may not prevent the progression of the cutaneous lesions [12].

There is also a paucity of guidelines regarding management owing to the rarity of this condition. Intravenous or LMWH, vitamin K, and/or fresh frozen plasma are often administered [2,8]. Prostacyclin had been successfully used for the treatment of WISN due to its antiplatelet aggregate and potent vasodilator effects [13]. Monoclonal antibody-purified concentrates of protein C is an effective therapy in patients with protein C deficiency [14]. Corticosteroids are not cited among adjunctive treatments. Their anti-inflammatory and immunosuppressive benefit is counterbalanced by their potential procoagulant effect that typically precludes steroid use in prothrombotic states. Our patient had a suspected associated systemic hypersensitivity reaction and was prescribed dexamethasone that likely contributed to the reversal of the skin lesions. It is uncertain if corticosteroids play a role in the treatment of WISN. However, it likely adds to the resolution of the skin findings if associated dermatitis or vasculitis is considered. Surgical treatment, including debridement and amputation, is required in approximately half of the cases [6].

Unlike warfarin, non-VKA oral anticoagulant drugs approved by the Food and Drug Administration agency such as dabigatran, rivaroxaban, and apixaban do not frequently need laboratory monitoring and have a non-inferior if not safer bleeding risk profile. Their use has been employed in WISN with case reports validating the use of rivaroxaban as an alternate anticoagulant [15,16]. Our patient was discharged on apixaban as current data suggest a low risk of major bleeding associated with apixaban versus rivaroxaban [17].

## Conclusions

Despite adequate enoxaparin bridging therapy, maintaining a low leading dose of warfarin, and a lack of clear drug interactions, our patient presented with cutaneous findings consistent with WISN. Physicians must maintain a high clinical suspicion for this rare but fatal disease even if proper bridging therapy has occurred because immediate discontinuation of warfarin is essential for avoiding significant morbidity and mortality. If an associated drug hypersensitivity reaction is suspected, steroids may also aid in treatment.
